# Tranexamic acid protects human dermal fibroblasts from D-galactose-induced senescence via the GPR30/MAPK pathway

**DOI:** 10.1080/07853890.2026.2663263

**Published:** 2026-04-30

**Authors:** Yanyan Lin, Yaling Wang, Wanjing Wang, Zhili Deng, Yiya Zhang, Yiran Peng, Jin Tang, Ji Li, Chuchu Huang, Dan Jian

**Affiliations:** ^a^ Department of Dermatology, Xiangya Hospital, Central South University, Changsha, China; ^b^ National Clinical Research Center for Geriatric Disorders, Xiangya Hospital, Central South University, Changsha, China; ^c^ Hunan Key Laboratory of ageing Biology, Xiangya Hospital, Central South University, Changsha, China; ^d^ Department of Dermatology, Shanghai Sixth People’s Hospital Affiliated to Shanghai Jiao Tong University School of Medicine, Shanghai, China

**Keywords:** Tranexamic acid, cellular senescence, skin ageing, GPR30, oxidative stress, MAPK

## Abstract

**Background:**

Tranexamic acid (TXA) is widely used for pigmentary disorders, but its anti-ageing potential remains unclear. This study aimed to evaluate whether topical 3% TXA improves early periorbital wrinkles in women with facial melasma and to investigate whether TXA protects human dermal fibroblasts from D-galactose-induced senescence *via* the GPR30/MAPK pathway.

**Methods:**

Fifty women with melasma were randomized to 3% TXA serum plus moisturizer or moisturizer alone for 8 weeks, with follow-up to week 12. Periorbital wrinkles were graded using a modified Fitzpatrick Wrinkle Scale (MFWS). Separately, D-gal-induced senescence in HDFs was assessed *via* viability, SA-β-gal activity, senescence markers, ROS, antioxidant enzymes, SASP/ECM gene expression, and MAPK activation. GPR30 involvement was examined using antagonist G15, shRNA knockdown, and molecular docking.

**Results:**

Topical TXA produced significantly greater MFWS reductions versus moisturizer alone at weeks 4, 8, and 12, with benefit persisting post-treatment. In HDFs, TXA preserved viability, reduced SA-β-gal positivity, attenuated p21/p16, restored Lamin B1, decreased ROS, and rescued antioxidant activities. TXA downregulated IL-6, IL-8, MMP1, and MMP3, and suppressed D-gal-induced ERK, JNK, and p38 phosphorylation. These effects were weakened by G15 or GPR30 knockdown; docking supported a stable TXA-GPR30 interaction.

**Conclusions:**

TXA showed clinical anti-wrinkle activity in melasma patients and protected HDFs from D-gal-induced senescence, partly *via* GPR30-dependent modulation of oxidative stress, SASP/ECM expression, and MAPK signalling. TXA is a promising candidate for skin ageing intervention.

## Introduction

Cellular senescence is a hallmark of ageing and contributes to the functional decline of multiple organs [1]. In skin, the gradual accumulation of senescent cells—particularly dermal fibroblasts—is increasingly recognized as a key driver of age-related phenotypes, including fine wrinkling, loss of elasticity and impaired barrier function [2]. Senescent fibroblasts display a stable cell-cycle arrest, a senescence-associated secretory phenotype (SASP) rich in pro-inflammatory and matrix-degrading factors, and profound alterations in extracellular matrix (ECM) homeostasis [3,4]. Experimental models using D-galactose (D-gal) to induce oxidative stress–driven senescence in human dermal fibroblasts (HDFs) recapitulate many of these features and are widely used to dissect the molecular mechanisms underpinning skin ageing [5,6].

Skin ageing is shaped by intrinsic processes, such as hormonal changes and replicative exhaustion, as well as extrinsic insults, most notably chronic ultraviolet exposure and pollution, with oxidative stress mediated by reactive oxygen species (ROS) representing a common downstream driver [7,8]. These factors converge on pathways involving excessive ROS production, mitochondrial dysfunction and activation of stress-responsive kinases, which further promote fibroblast senescence and ECM breakdown [9,10]. Consequently, strategies that limit oxidative stress, blunt SASP signalling or preserve fibroblast function are being actively explored as potential interventions to delay or reverse cutaneous ageing.

Tranexamic acid (TXA) is a synthetic lysine analogue originally developed as an antifibrinolytic agent [11]. In dermatology, topical and oral TXA have become established options for treating melasma and other dyschromias, and are thought to exert anti-inflammatory, anti-oxidative and vasculature-stabilizing effects in addition to inhibiting plasmin activity [11,12]. Experimental work in ageing mouse models has further suggested that TXA can extend lifespan and ameliorate age-associated cutaneous and systemic changes [13–15]. However, its anti-ageing potential and the underlying mechanisms remain poorly defined in human skin, particularly at the level of dermal fibroblasts.

G protein–coupled receptor 30 (GPR30, also termed GPER1) is a membrane G protein–coupled receptor expressed in multiple tissues, including skin cell types such as keratinocytes and fibroblasts [16]. Activation of GPR30 can modulate intracellular calcium, cyclic AMP and mitogen-activated protein kinase (MAPK) signalling cascades, and has been linked to the regulation of oxidative stress responses, inflammatory signalling and tissue remodelling in cardiovascular, immune–inflammatory, endocrine–metabolic, nervous and skeletal systems [17–22]. In skin-related models, GPR30 has been implicated in cutaneous homeostasis, innate immune responses and tissue protection, and pharmacologic activation of GPR30 has been reported to attenuate UV-induced epidermal stem cell damage [16,23–25]. However, its role in skin ageing and its potential involvement in TXA-related signalling have not been fully elucidated.

With the growing clinical use of TXA for pigmentary disorders, its incidental anti-ageing effects warrant systematic investigation. In a randomized clinical trial originally designed to evaluate topical TXA for facial melasma, we observed that periorbital fine lines appeared to improve more prominently in the TXA group than in the moisturizer-alone group. Building on this observation, the present study had two aims: first, to quantitatively characterize the impact of topical 3% TXA on early periorbital wrinkles in women with facial melasma using standardized photographic assessment and wrinkle scoring; and second, to determine whether TXA protects HDFs from D-gal–induced senescence *via* GPR30-dependent modulation of oxidative stress, SASP/ECM-related gene expression and MAPK signalling. Through this combined clinical and mechanistic approach, we sought to clarify the anti-ageing potential of TXA and provide a rationale for its use in the prevention and treatment of skin ageing.

## Methods

### Ethics statement

**Figure 1.**
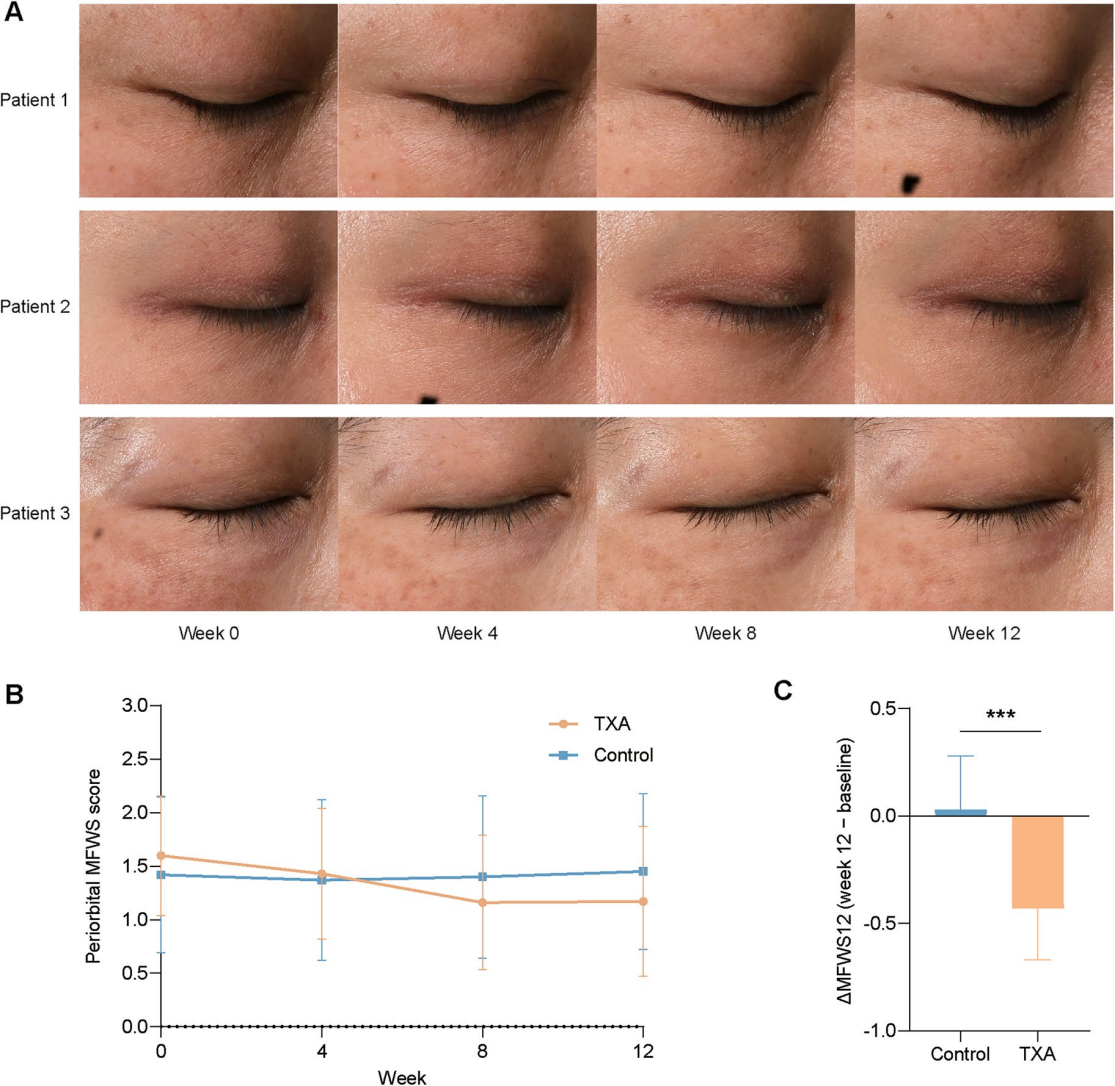
Topical 3% tranexamic acid improves periorbital wrinkles in women with facial melasma. (A) Serial VISIA^®^ facial photographs from three patients in the TXA + moisturizer group at baseline (week 0) and weeks 4, 8 and 12, illustrating a progressive softening of periorbital fine lines over time. (B) Mean periorbital Modified Fitzpatrick Wrinkle Scale (MFWS) scores in the TXA and moisturizer-alone groups at baseline and weeks 4, 8 and 12 (mean ± SD, *n* = 25 per group). (C) Change in periorbital MFWS from baseline to week 12 (ΔMFWS12); negative values indicate wrinkle reduction. Data are mean ± SD; ***p < 0.001 versus moisturizer alone (independent-samples t-test).

This study was approved by the Medical Ethics Committee of Xiangya Hospital, Central South University (Approval No. 1.1 20210327) and conducted in accordance with the ethical principles outlined in the 1964 Declaration of Helsinki and its subsequent amendments. Written informed consent was obtained from all participants, or for minors, from their parents or legal guardians. The study protocol, including the collection of skin samples and clinical images, was reviewed and approved by the Ethics Committee of Xiangya Hospital, Central South University. All clinical images of patients shown in Figure 1 were obtained with written consent for publication from the Department of Dermatology at Xiangya Hospital, Central South University, China. The study’s purpose, procedures, risks, and benefits were clearly explained to participants, and all voluntarily consented to participate and allow the use of their samples.

### Exploratory clinical evaluation of periorbital wrinkles

This exploratory analysis was based on a randomized, moisturizer-controlled trial of topical tranexamic acid (TXA) for facial melasma conducted at the Department of Dermatology, Xiangya Hospital, Central South University (Changsha, China). Between June and December 2021, fifty women were enrolled and randomly assigned (1:1) to receive either 3% TXA serum plus a basic moisturizer (TXA group) or the same moisturizer alone (control group). The allocated products were applied twice daily to the entire face for 8 weeks, followed by observation without further treatment to week 12.

Inclusion criteria were: female sex; age 18–60 years; clinical diagnosis of facial melasma involving the face; Fitzpatrick skin phototypes II–IV; and willingness to avoid other topical or systemic depigmenting agents, anti-wrinkle products or cosmetic procedures during the study period. Exclusion criteria included pregnancy or lactation; a history of thromboembolic disease or coagulation disorders; current use of systemic anticoagulants; known hypersensitivity to TXA or any component of the study products; active inflammatory or infectious dermatoses on the face; and clinically significant hepatic, renal, cardiovascular, haematologic or other systemic disease judged by the investigators to interfere with study participation. All participants provided written informed consent, and the protocol was approved by the Ethics Committee of Xiangya Hospital (Approval No. 1.1 20210327).

### Assessment of periorbital wrinkles

At baseline (week 0) and at weeks 4, 8 and 12, standardized facial photographs were acquired using a VISIA^®^ complexion analysis system (Canfield Scientific, Fairfield, NJ, USA) under controlled lighting and positioning. Two board-certified dermatologists, blinded to treatment allocation and visit order, independently graded periorbital wrinkles on each image using a modified Fitzpatrick Wrinkle Scale (MFWS). For each photograph, the final MFWS value was defined as the mean of the two raters’ scores. Inter-rater reliability of MFWS at each visit was quantified using a two-way random-effects, absolute-agreement, single-measures intraclass correlation coefficient (ICC[1,2]) with 95% confidence intervals.

### Statistical analysis of wrinkle outcomes

Clinical wrinkle data were analyzed using SPSS 26.0 (IBM Corp., Armonk, NY, USA). Changes in periorbital wrinkles from baseline were calculated as ΔMFWS4, ΔMFWS8 and ΔMFWS12 (follow-up MFWS minus baseline MFWS), with negative values indicating improvement. Between-group differences in ΔMFWS were assessed using independent-samples t-tests. In addition, analysis of covariance (ANCOVA) was performed with MFWS at weeks 4, 8 and 12 as the dependent variable, treatment group (TXA vs. control) as a fixed factor and baseline MFWS as a covariate, to obtain baseline-adjusted between-group differences and estimated marginal means with 95% confidence intervals. All tests were two-sided, and *p* < 0.05 was considered statistically significant.

### Reagents

Tranexamic Acid (purity >98%) was supplied by Central Inspection Institute. D-Galactopyranose was obtained from Hushi. Antibodies against p21, p16, pRb, Rb, Lamin B1, phospho-JNK (p-JNK), JNK, phospho-ERK (p-ERK), ERK, phospho-p38 (p-38), and p38 were supplied by Cell Signaling Technology. GPR30 antibodies were obtained from Abcam. Detailed information on all primary and secondary antibodies, including manufacturer, catalog number and working dilution, is provided in Supplementary Table S1.

### Cell culture

Primary human dermal fibroblasts (HDFs) were isolated and cultured from the circumcised foreskins of healthy male donors aged 5–20 years, with informed consent and protocol approval from the Clinical Research Ethics Committee of Xiangya Hospital, Central South University. 293 T cells were obtained from ATCC. Cells were maintained in Dulbecco’s modified Eagle medium (DMEM) supplemented with 10% fetal bovine serum (FBS), penicillin (100 U/mL), and streptomycin (100 μg/mL) at 37 °C in a humidified incubator with 5% CO2.

### Cell viability assay

Cell viability was assessed using a Cell Counting Kit-8 (CCK-8, Beyotime Biotechnology). Briefly, cells were seeded into 96-well plates at a density of 3 × 10^3 cells per well and pretreated for 24 h before TXA or D-gal treatment. Afterward, CCK-8 solution was added to each well and incubated for 2–4 h. Absorbance was measured at 450 nm using a spectrophotometer to assess cell proliferation. Based on these preliminary dose–response experiments, 200 µg/mL TXA was selected as the working concentration and was used in all subsequent *in vitro* assays.

### SA-β-gal staining

SA-β-gal staining was performed using a kit from Beyotime Biotechnology. HDFs were seeded in six-well plates and stained according to the kit instructions. Cells were incubated in a 37 °C dry incubator (without CO2). SA-β-gal-positive cells were identified by their blue color and counted under a phase-contrast microscope.

### Western blotting

HDFs were lysed in cold RIPA buffer containing protease and phosphatase inhibitors (Thermo Fisher Scientific). Lysates were centrifuged at 12,000 g for 15 min, and the supernatants were collected. Protein concentration was determined using a BCA protein assay kit (Beyotime Biotechnology). Proteins (30 μg) were separated by 8%, 10%, or 12% SDS-PAGE, transferred to PVDF membranes, and blocked with 5% milk for 1 h at room temperature. Membranes were incubated with primary antibodies overnight at 4 °C. After washing with TBST, secondary antibodies were applied and incubated for 1 h at room temperature. Membranes were visualized using ECL reagent (Bio-Rad Laboratories) and analyzed using ImageJ software.

### Quantitative RT-PCR (qRT-PCR) assay

Gene primers were synthesized by Sangon Biotech (primer sequences listed in Table 1). Total RNA was extracted using TRIzol (Thermo Fisher Scientific) and reverse-transcribed using the PrimeScript^™^ RT reagent Kit with gDNA Eraser (Takara). qPCR was performed using iTaq^™^ Universal SYBR Green Supermix (Bio-Rad) on a LightCycler 96 system (Roche). All primer sequences used are provided in Table S2.

**Table 1.** Baseline demographic and clinical characteristics of the study participants.

Characteristic	Moisturizer alone (*n* = 25)	TXA + moisturizer (*n* = 25)	P value
Age, y	36.72 ± 1.07	36.72 ± 1.01	1.000
Fitzpatrick skin phototype, n (%)			0.934
III	5 (20.0)	4 (16.0)	
IV	19 (76.0)	20 (80.0)	
V	1 (4.0)	1 (4.0)	
Baseline periorbital MFWS, mean ± SD	1.42 ± 0.73	1.60 ± 0.56	0.332

Data are presented as mean ± SD or n (%), as appropriate. P values are from independent-samples t tests for continuous variables and χ^2^ tests for categorical variables.

### Intracellular ROS level

A reactive oxygen species (ROS) detection kit (Beyotime Biotechnology) was used to measure ROS levels. After completing the treatment incubation, cells plated in 6-well plates were washed with PBS. Then, 2 mL of dichloro-dihydro-fluorescein diacetate (DCFH-DA, 10 µM) diluted in basal medium was added to each well, and the cells were incubated for an additional 20 min. After washing the cells 2–3 times with basal medium, 1 mL of PBS was added. Fluorescence was observed and photographed using an inverted fluorescence microscope.

### Analysis of anti-oxidative capacity

Following treatment, HDFs were collected and analyzed according to the manufacturer’s instructions. Superoxide dismutase (SOD) and catalase (CAT) activities were measured using the Total SOD Assay Kit with WST-8 and the Catalase Assay Kit (both from Beyotime Biotechnology). Lipid oxidative damage, indicated by malondialdehyde (MDA) levels, was assessed using a Lipid Oxidation (MDA) Assay Kit (Beyotime Biotechnology).

### Molecular docking

The 3D structure of the GPR30 protein target was downloaded in PDB format from the PDB database (https://www.rcsb.org/), while the SDF structure of tranexamic acid was obtained from PubChem and converted to a PDB file using Chem3D. AutoDock Tools 1.5.6 was used to prepare the protein by removing water and adding hydrogens, converting both the protein and ligand to pdbqt format. Molecular docking was then performed using AutoDock Vina, and the results were visualized with Pymol software.

### RNA interference

Short hairpin RNA (shRNA) targeting GPR30 was encoded in pLent-U6-GFP-Puro lentiviruses, constructed by ViGene Biosciences (Shandong, China). The following sequences were used: shRNA1, GATCCGCTCCCTCATTGAGGTGTTCAATTCAAGAGATTGAACACCTCAATGAGGGAGTTTTTT; shRNA2, GATCCGCGCTCCCTGCAAGCAGTCTTTTTCAAGAGAAAAGACTGCTTGCAGGGAGCGTTTTTT; and shRNA3, GATCCGTCCTTCTCCTCTTTAACTCTTCAAGAGAGAGTTAAAGAAGAAGGATTTTTTA. HDFs were infected with lentiviruses (1 × 10^8 TU/mL) and cultured in complete DMEM containing 400 ng/mL of puromycin for 2 weeks to establish stable clones. GPR30 knockdown was confirmed, and these cells (shGPR30) were used in further experiments. Empty vectors were used as controls.

### Statistical analysis

*In vitro* data were analyzed with GraphPad Prism 9.0 (GraphPad Software, San Diego, CA, USA). For comparisons between two groups, unpaired Student’s t-tests were used; for multiple groups, one-way ANOVA with appropriate post hoc tests was applied. Results are expressed as mean ± standard deviation (SD).

## Results

### Topical TXA reduces early periorbital wrinkles in women with facial melasma

All 50 randomized women (25 per group) completed the 8-week treatment and the week-12 follow-up. Baseline demographic and pigment-related characteristics were comparable between the two groups (Table 1). Most participants had Fitzpatrick skin phototypes III–IV, and baseline periorbital MFWS scores were modest and similar in the TXA and control groups (1.60 ± 0.56 vs 1.42 ± 0.73, *p* = 0.332), indicating a comparable degree of early periorbital wrinkling at study entry.

Inter-rater reliability for MFWS grading was high at all visits. Single-measures ICC values were 0.920 (95% CI 0.859–0.955) at baseline, 0.886 (0.791–0.937) at week 4, 0.913 (0.844–0.951) at week 8 and 0.917 (0.859–0.952) at week 12 (all *p* < 0.001), reflecting good to excellent agreement between the two dermatologists.

Periorbital MFWS decreased over time in both groups, but improvements were consistently greater with TXA (Table 2 and Figure 1B). At week 4, the mean change from baseline (ΔMFWS4) was −0.17 ± 0.19 in the TXA group versus −0.05 ± 0.10 in the moisturizer-only group (*p* = 0.007). At week 8, ΔMFWS8 was −0.44 ± 0.23 with TXA compared with −0.02 ± 0.19 in controls (*p* < 0.001). By week 12, the TXA group maintained a clear reduction in wrinkle scores (ΔMFWS12 = −0.43 ± 0.24), whereas the control group had essentially returned to baseline (ΔMFWS12 = 0.03 ± 0.25; *p* < 0.001). This net treatment effect at week 12 is summarized in Figure 1C, and representative VISIA facial photographs from a TXA-treated patient qualitatively illustrate the visible reduction in periorbital fine lines from baseline to week 12 (Figure 1A).where the more negative ΔMFWS12 in the TXA group illustrates the greater reduction in periorbital wrinkles compared with moisturizer alone.

**Table 2.** Changes in periorbital MFWS from baseline to weeks 4, 8, and 12.

Outcome	Moisturizer alone (*n* = 25)	TXA + moisturizer (*n* = 25)	P value
ΔMFWS 4	−0.05 ± 0.10	−0.17 ± 0.19	0.007
ΔMFWS 8	−0.02 ± 0.19	−0.44 ± 0.23	<0.001
ΔMFWS 12	0.03 ± 0.25	−0.43 ± 0.24	<0.001

ΔMFWS was calculated as follow-up MFWS minus baseline MFWS; negative values indicate wrinkle reduction. Data are mean ± SD.

ANCOVA analyses adjusting for baseline MFWS confirmed that these between-group differences were not attributable to baseline imbalance (Table 3). After adjustment, mean periorbital MFWS values were significantly lower in the TXA group than in the control group at all follow-up visits: 1.34 ± 0.03 versus 1.46 ± 0.03 at week 4 (*p* = 0.006), 1.07 ± 0.04 versus 1.49 ± 0.04 at week 8 (*p* < 0.001) and 1.08 ± 0.06 versus 1.60 ± 0.06 at week 12 (*p* < 0.001). Taken together, these clinical data indicate that topical 3% TXA plus moisturizer produces a robust and sustained reduction in early periorbital wrinkles compared with moisturizer alone, which prompted us to explore the underlying anti-ageing mechanisms *in vitro*.

**Table 3.** Baseline-adjusted periorbital MFWS at weeks 4, 8, and 12 (ANCOVA).

Time point	Moisturizer alone (adjusted mean ± SE)	TXA + moisturizer (adjusted mean ± SE)	P value
Week 4	1.462 ± 0.030	1.338 ± 0.030	0.006
Week 8	1.492 ± 0.043	1.068 ± 0.043	<0.001
Week 12	1.596 ± 0.055	1.084 ± 0.055	<0.001

Adjusted means ± standard error (SE) are from ANCOVA models with baseline MFWS as a covariate and treatment group as a fixed factor. P values are for between-group differences at each time point.

### TXA inhibits D-galactose-induced senescence of HDFs

**Figure 2.**
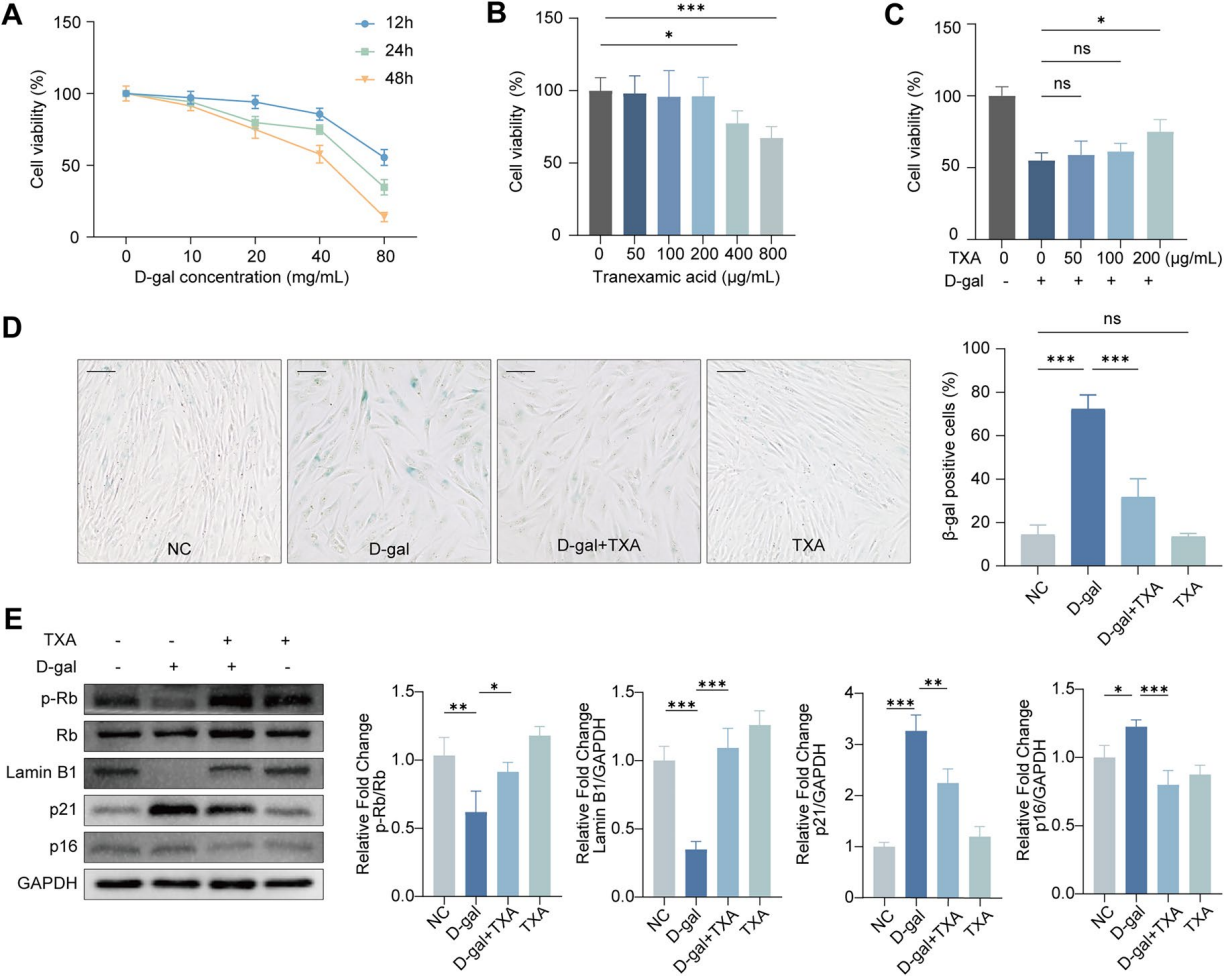
TXA attenuates D-galactose-induced senescence in HDFs. (A) Effects of different concentrations of D-gal on HDF proliferation at 12, 24, and 48 h, assessed by CCK-8 assay. (B) Effects of TXA (50–800 μg/mL) alone on HDF viability. (C) Effects of TXA (50–200 μg/mL) on HDF viability in the presence of D-galactose; 200 μg/mL was used in subsequent experiments. (D) SA-β-gal staining of HDFs. Scale bar = 100 μm. (E) Western blot analysis of pRb, Rb, Lamin B1, p21, and p16. Data are presented as mean ± SD. *p < 0.05, **p < 0.01, ***p < 0.001.

We first examined the anti-senescent effects of TXA in HDFs, including cell viability, SA-β-gal positivity, and canonical senescence-associated protein markers (Figure 2). Senescent cells show a marked reduction in proliferative capacity, so we first assessed the effects of D-galactose (D-gal) on HDF viability at 12, 24 and 48 h using the CCK-8 assay. D-gal suppressed HDF proliferation in a concentration- and time-dependent manner, and 40 mg/mL D-gal, which reduced cell viability to approximately 50% after 48 h, was selected as the standard senescence-inducing condition (Figure 2A). To define a safe and effective TXA range, we next examined TXA alone over a series of doses and found that 50–200 µg/mL did not significantly affect HDF proliferation or viability (Figure 2B). By contrast, pretreatment with increasing TXA concentrations (50–200 µg/mL) progressively attenuated D-gal–induced growth inhibition, with 200 µg/mL providing the most consistent protection (Figure 2B,C). On this basis, 200 µg/mL was chosen as the working concentration for subsequent experiments. We then evaluated whether TXA could mitigate D-gal-induced senescence in HDFs by examining classical senescence markers. SA-β-gal staining demonstrated a pronounced reduction in senescent cells in cultures exposed to both D-gal and TXA compared with those treated with D-gal alone (Figure 2D). Consistently, Western blotting showed that TXA markedly decreased p21, p16, and pRb/Rb expression while restoring Lamin B1 levels (Figure 2E). Taken together, these data indicate that TXA effectively inhibits D-galactose-induced senescence in HDFs.

### Effect of TXA on inhibiting the expression of SASP in HDFs

**Figure 3.**
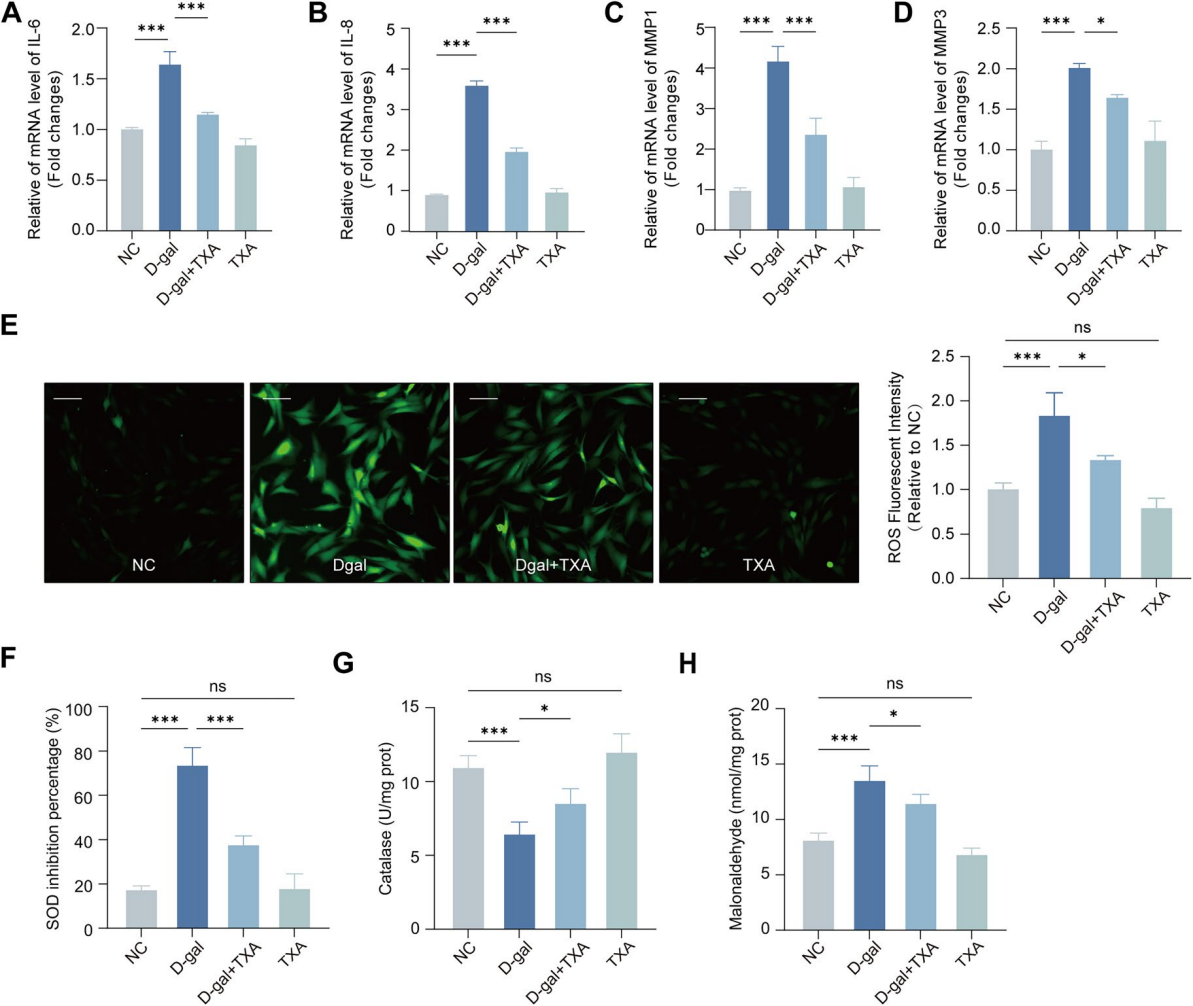
TXA suppresses SASP-related gene expression and oxidative stress in senescent HDFs. (A–D) Relative mRNA expression of IL-6, IL-8, MMP1, and MMP3. (E) Intracellular ROS levels. (F–H) SOD activity, CAT activity, and MDA content. Data are presented as mean ± SD. *p < 0.05, **p < 0.01, ***p < 0.001.

We next evaluated two closely related downstream consequences of cellular senescence, namely SASP/ECM-related transcriptional changes and oxidative stress status (Figure 3). In D-gal–treated HDFs, TXA markedly reduced the D-gal-induced upregulation of IL-6 and IL-8 and also lowered the expression of the ECM-associated metalloproteinase genes MMP1 and MMP3 (Figure 3A–D). From this pattern, it follows that TXA attenuates SASP-related inflammation and reduces the matrix-degrading potential at the level of gene expression.

### TXA inhibits ROS production and potentiates the antioxidant capacity of HDFs

The most likely mechanism of D-gal-induced senescence is induced oxidative stress damage. We thus explored whether TXA could inhibit the effect of D-gal by reducing the oxidative stress. The DCFH-DA probe was used to detect intracellular ROS by fluorescence microscopy, we found that TXA significantly reduced ROS generation in HDFs compared to the D-gal-treated group (Figure 3E). Next, we analyzed the activities of antioxidant enzyme system. The results indicated that TXA significantly counteracted the D-gal-induced inhibition of SOD activity and increased CAT activity (Figure 3F,G). Furthermore, TXA significantly reduced MDA levels,suggesting protection against D-gal-induced membrane damage (Figure 3H). Overall, these results support that TXA is effective in protecting HDFs from D-gal-induced oxidative damage by suppressing oxidative stress, reducing ROS production, and enhancing antioxidant defense systems.

### TXA positively regulates GPR30 and inhibits the activation of MAPKs in D-gal-induced senescence

**Figure 4.**
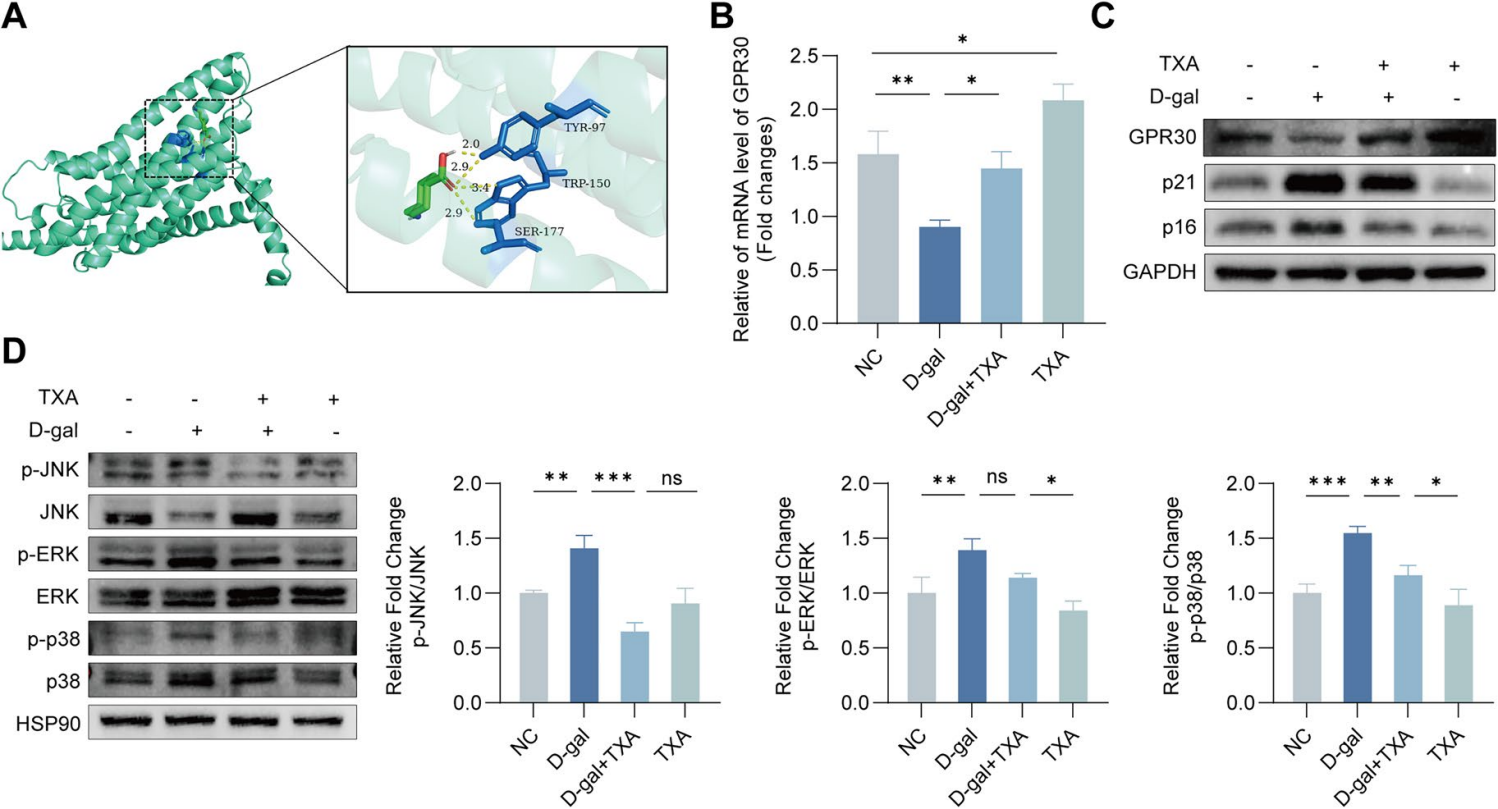
TXA regulates GPR30 expression and MAPK signaling in senescent HDFs. (A) Binding mode of TXA to GPR30. (B) GPR30 protein expression. (C) GPR30 mRNA expression. (D) Western blot analysis of p-JNK, JNK, p-ERK, ERK, p-p38, and p38. Data are presented as mean ± SD. *p < 0.05, **p < 0.01, ***p < 0.001.

**Figure 5.**
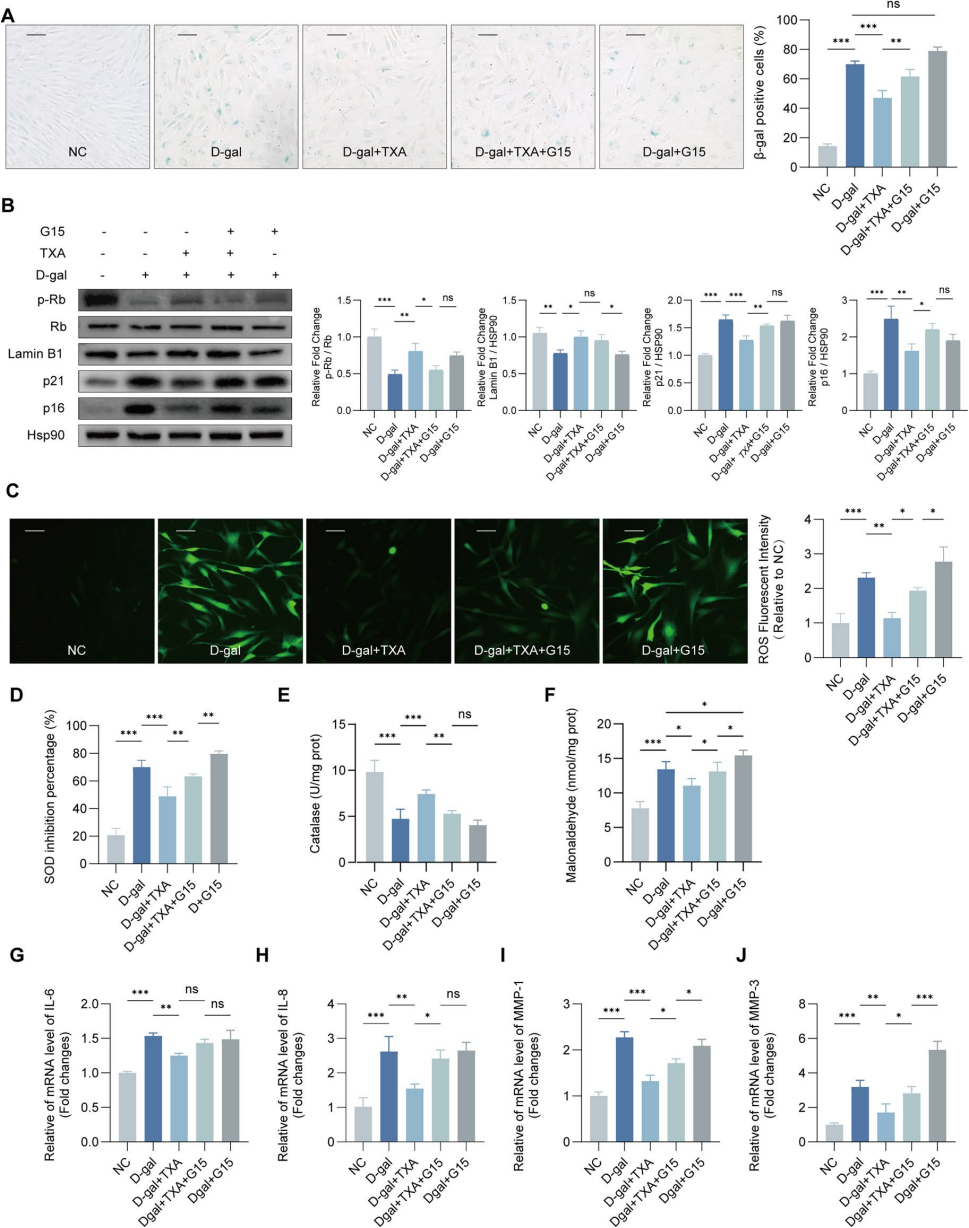
G15 attenuates the anti-aging-related effects of TXA by inhibiting GPR30 activity. (A) SA-β-ga l staining of HDFs. Scale bar = 100 μm.(B) Western blot analysis of pRb, Rb, Lamin B1, p21, and p16 expression. (C) Representative fluorescence images showing intracellular ROS levels in HDFs detected b y DCFH-DA. Scale bar = 100 μm. (D) SOD activity. (E) CAT activity. (F) MDA content. (G–J) Relative mRNA expression of IL-6, IL-8, MMP1, and MMP3 determined by qRT-PCR. Data are presented as mean ± SD. *p < 0.05, **p < 0.01, ***p < 0.001.

**Figure 6.**
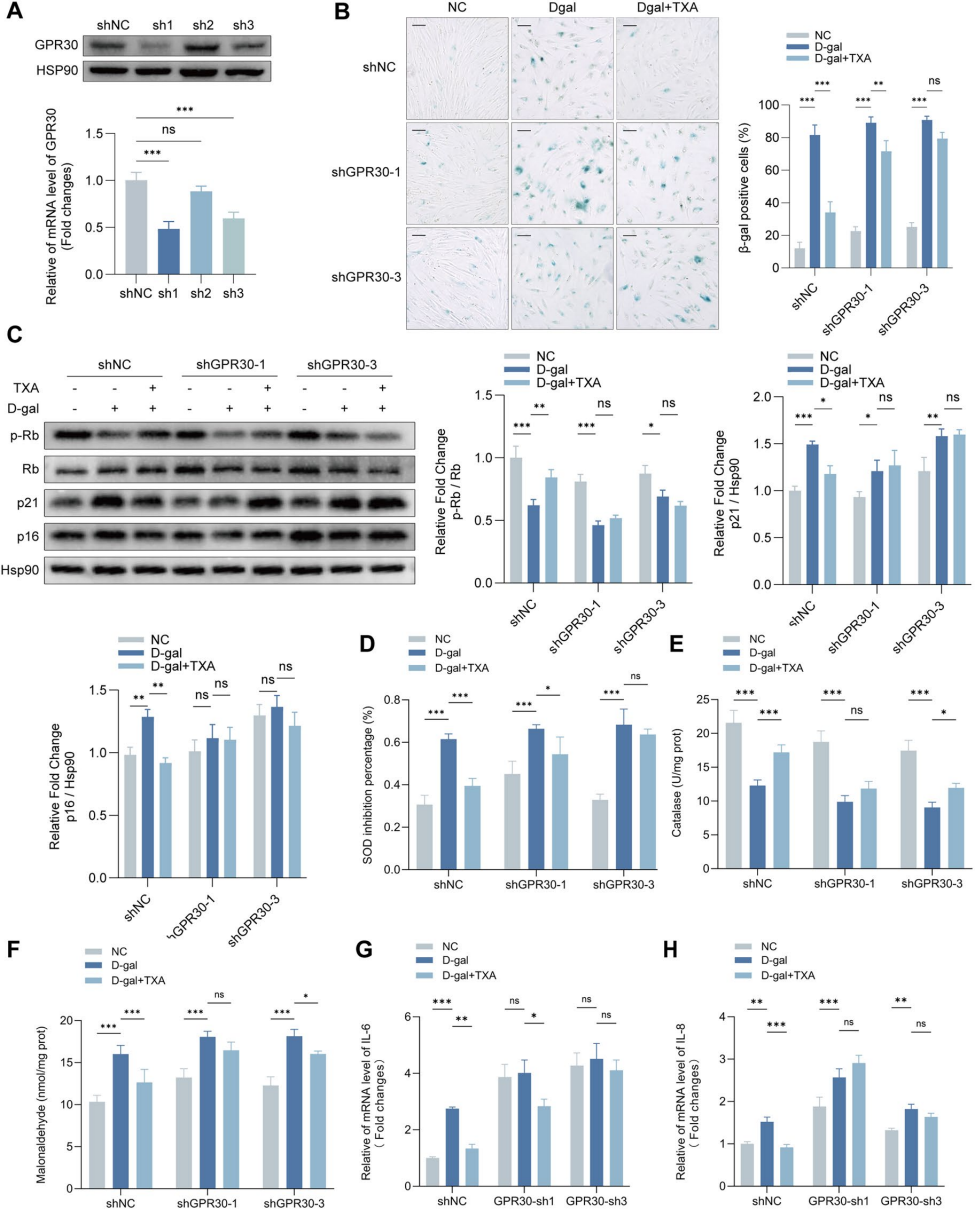
GPR30 knockdown attenuates the protective effects of TXA in HDFs. (A) Validation of GPR30 knockdown by Western blotting and qRT-PCR. (B) SA-β-gal staining of GPR30-knockdown HDFs. Scale bar = 100 μm. (C) Western blot analysis of pRb, Rb, p21, and p16. (D–F) SOD activity, CAT activity, and MDA content. (G, H) Relative mRNA expression of IL-6 and IL-8. shGPR30-1 and shGPR30-3 denote two independent shRNAs targeting GPR30; shNC denotes the non-targeting control. Data are presented as mean ± SD. *p < 0.05, **p < 0.01, ***p < 0.001.5, **p < 0.01, ***p < 0.001.

**Figure 7.**
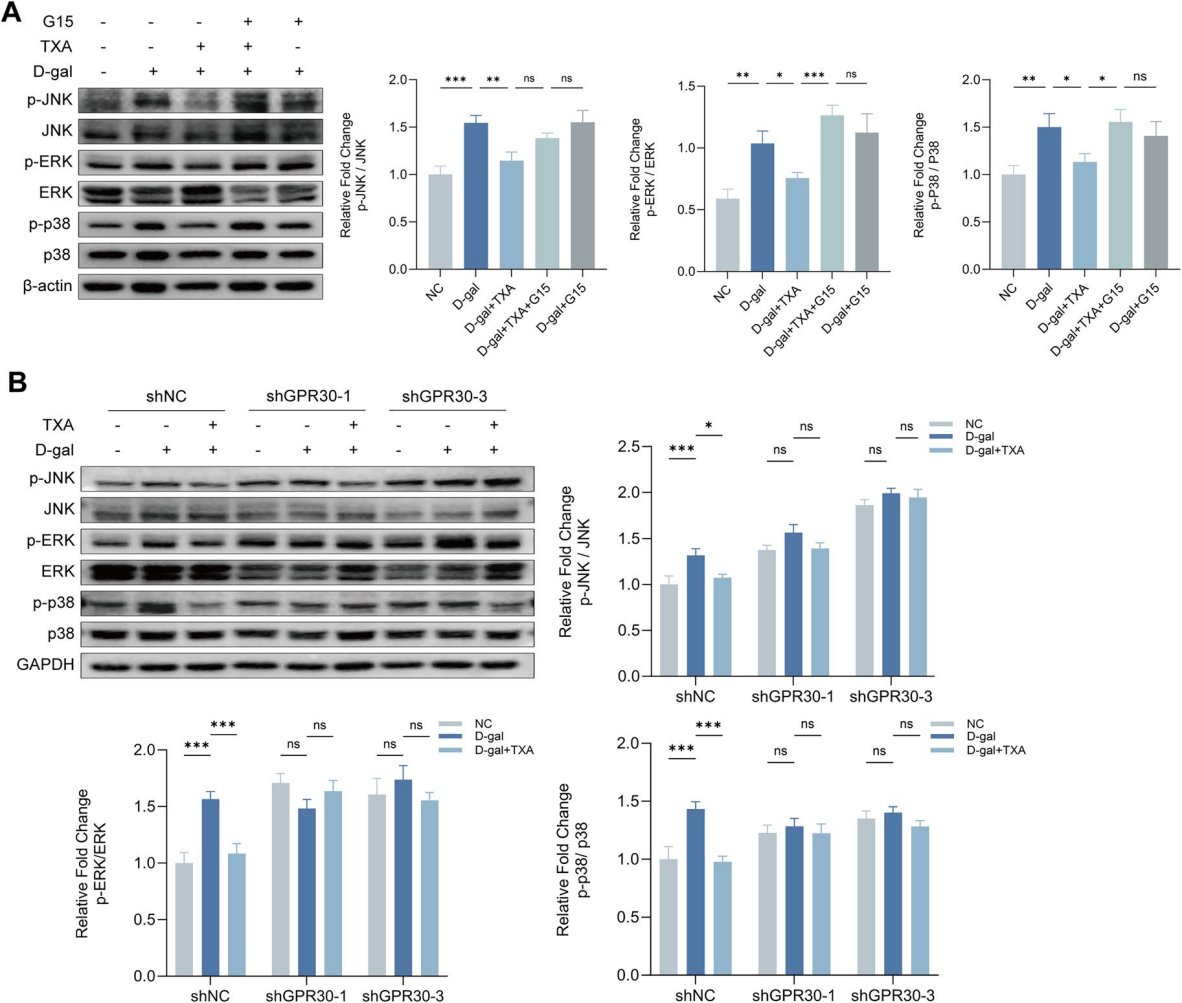
Reduction of GPR30 activity or expression weakens TXA-mediated suppression of MAPK signa ling.(A–B) Western blot analysis of p-JNK, JNK, p-ERK, ERK, p-p38, and p38 expression after pharmacologic inhibition or genetic knockdown of GPR30. Data are presented as mean ± SD. *p < 0.05, **p < 0.01, ***p < 0.001.

To investigate the underlying mechanism, we then examined GPR30 expression and MAPK pathway activation, followed by pharmacologic inhibition and genetic knockdown experiments (Figures 4–7). Initially, molecular docking technology was employed to probe the interaction between tranexamic acid and GPR30. The resultant binding energy was −5.4 kcal/mol, suggestive of a robust interaction between the two entities (Figure 4A). The influence of TXA on both the protein and mRNA levels of GPR30 in D-galactose-induced senescent HDFs was investigated, with findings indicating that TXA treatment upregulates GPR30 expression (Figure 4B,C). Moreover, Western blot analyses demonstrate that TXA inhibits the phosphorylation of key MAPK pathway proteins, including JNK, ERK, and p38 (Figure 4D), suggesting a downregulation of this pathway’s activation in senescence. Collectively, the data underscore TXA’s role in modulating GPR30 and impeding MAPK pathway activation during cellular senescence, presenting potential therapeutic implications for ageing and senescence-related disorders.

### G15 reverses the protective effect of TXA on HDFs senescence

Our study first demonstrated that TXA positively regulates GPR30 amidst D-galactose-induced senescence in HDFs. To investigate whether GPR30 inhibition could mitigate the effects of TXA, we incorporated the specific inhibitor G15 to determine the role of TXA in modulating GPR30 against HDFs senescence.Prior to TXA treatment, G15 was applied to inhibit GPR30, and subsequent evaluations showed that TXA’s anti-ageing benefits were reduced upon GPR30 inhibition, as indicated by increased SA-β-Gal activity and the upregulation of p16 and p21, coupled with decreased phosphorylation of pRb; however, Lamin B1 levels were not significantly altered (Figure 5A,B). A control group with D-gal and G15 was established to isolate G15′s specific effects, confirming that while GPR30 functions as a protective molecule against ageing, its inhibition does not exacerbate cellular senescence. These findings imply that GPR30’s suppression *via* G15 diminishes TXA’s senescence-combating efficacy. TXA alleviates cellular ageing by reducing D-gal-induced oxidative damage, but G15′s presence partially negates TXA’s inhibitory influence on ROS production (Figure 5C). Additionally, G15 notably decreases TXA’s protective effects on the activities of SOD and CAT and reverses the reduction of MDA levels promoted by TXA. When cells were treated with G15 alone prior to D-gal exposure, ROS production and SOD and CAT levels did not decrease further than in the D-gal only group, yet MDA levels increased, intensifying oxidative membrane damage (Figure 5D,E,F). This study further examines how G15 modulates TXA’s regulatory effects on inflammatory and matrix remodeling factors in D-gal-induced cellular senescence. The findings show that D-gal treatment significantly upregulates mRNA levels of IL-6, IL-8, MMP1, and MMP3, whereas TXA pre-treatment curtails these SASP factors’ expression. Inhibition of GPR30 *via* G15 reverses TXA’s suppression of IL-8, MMP1, and MMP3 transcription levels (Figure 5G,H,I,J).

### The protective effects of TXA are dependent on the normal level of expression of GPR30

To substantiate the impact of TXA on GPR30 regulation and its consequent effects on HDF ageing, we employed shRNA-GPR30-1 and 3 to knock down GPR30 for subsequent analyses (Figure 6A). Following this, we assessed the influence of TXA on senescence markers. The absence of GPR30 significantly nullified the protective effects of TXA during D-gal-induced senescence. In the shNC group, TXA markedly decreased SA-β-Gal activity, which was induced by D-gal. Nonetheless, the percentage of SA-β-Gal positive cells in the D-gal + TXA group saw only a 10% reduction compared to the D-gal group (Figure 6B), notably less than the nearly 50% reduction observed in the shNC group. Further analysis of cell cycle regulatory proteins indicated that in the shNC group, TXA mitigated the D-gal-induced activation of p16 and p21 and the reduction of p-Rb, signifying its anti-ageing potential. Intriguingly, in shGPR30-1 and shGPR30-3, TXA’s effect was partially or entirely inverted (Figure 6C). Additional scrutiny of TXA’s antioxidant capabilities revealed that in shNC cells, TXA significantly reduced D-gal-induced oxidative stress, as demonstrated by lowered SOD inhibition, heightened CAT activity, and reduced MDA levels. Conversely, in shGPR30-1 and shGPR30-3, the antioxidant properties of TXA were distinctly diminished (Figure 6D,E,F). The expression of IL-6 and IL-8 mRNA, upon analysis, showed that the absence of GPR30 also compromised the anti-inflammatory effects of TXA (Figure 6G,H). These observations align with the results obtained when GPR30 was inhibited using G15, indicating that TXA’s anti-ageing effects are potentially mediated by GPR30. Consistent with a GPR30-dependent mechanism, G15 treatment or GPR30 knockdown largely reversed the ability of TXA to reduce SA-β-gal positivity, lower ROS levels, restore SOD and CAT activities, and suppress IL-6, IL-8, MMP1 and MMP3 expression (Figures 5 and 6).

### Reduction of GPR30 activity or expression reverses TXA inhibition of D-gal-induced cellular senescence by activating MAPKs

To elucidate the mechanism by which TXA inhibits MAPK pathway activation in senescent conditions, particularly through its interaction with or influence on GPR30 expression, key proteins within the MAPK pathway were examined under GPR30 suppression. TXA was observed to diminish D-galactose-induced MAPK activation; however, this reduction was reversed with G15, a GPR30 inhibitor. Analysis of the D-gal + G15 group revealed that GPR30 inhibition alone did not synergize with D-gal to enhance MAPK activation during senescence. Nonetheless, it obstructed the D-gal-reduced MAPK activation, indicating TXA’s reliance on normal GPR30 activity within cells. Moreover, TXA significantly reduced D-gal-induced phosphorylation of JNK, ERK, and p38 in shNC, whereas in shGPR30-1 and shGPR30-3, the phosphorylation levels were intrinsically higher. These cells did not exhibit further phosphorylation with D-gal treatment, and the reduction in phosphorylation by TXA was notably limited (Figure 7A,B). These findings support the notion that TXA’s protective effects in D-gal-induced HDFs are largely dependent on intact GPR30 expression. Thus, modulation of the GPR30/MAPK pathway could be crucial to HDFs’ resistance to oxidative stress and ageing induced by D-gal.

## Discussion

**Figure 8.**
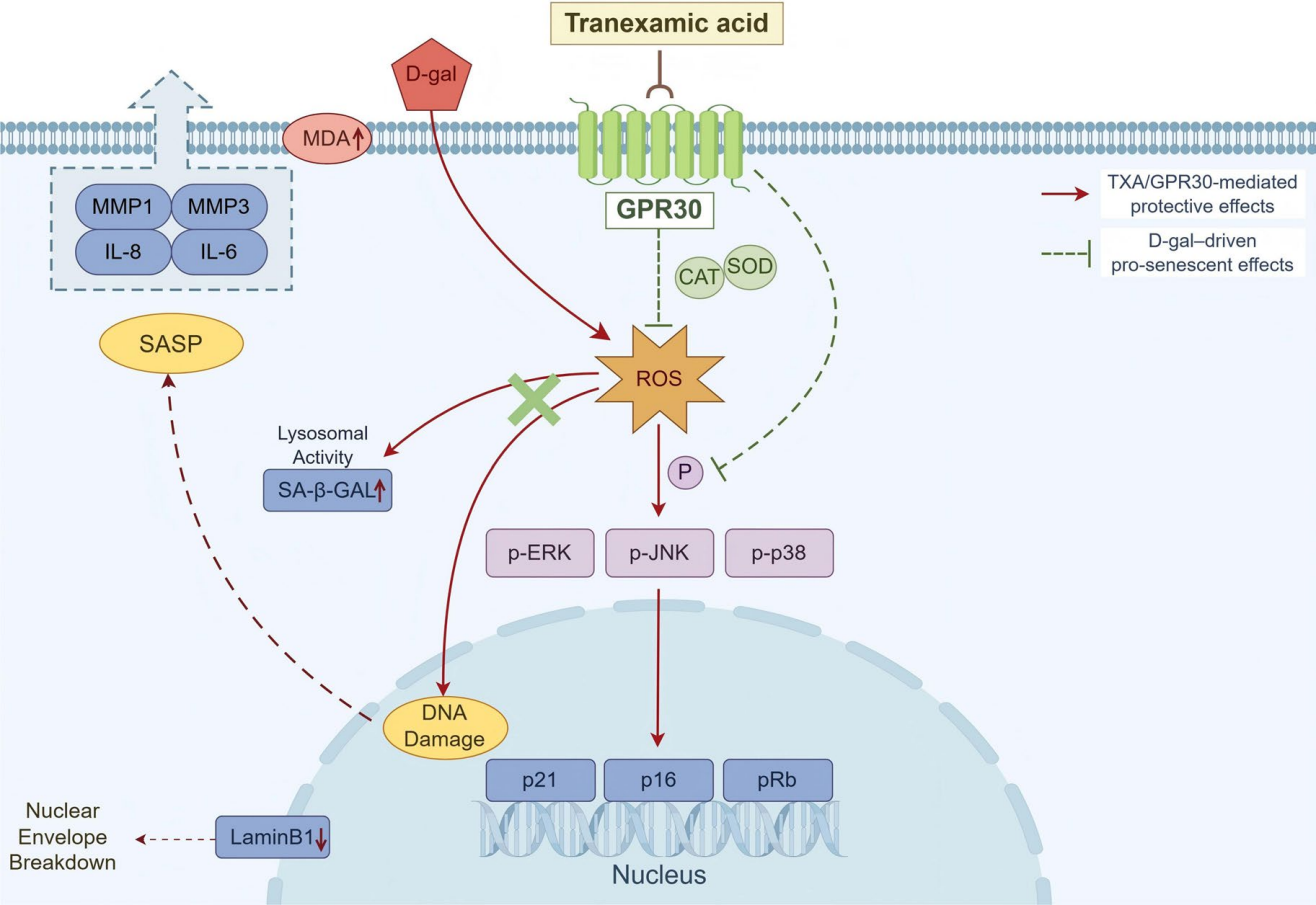
TXA protects human dermal fibroblasts from D-galactose–induced senescence *via* the GPR30/MAPK pathway.

With the growing understanding of ageing biology, the search for safe, mechanism-based strategies to delay skin ageing has become a central theme in dermatology [1,3,4]. In our previous randomized clinical study of topical tranexamic acid (TXA) for melasma, we incidentally noted that 3% TXA serum not only improved hyperpigmentation but also visibly softened fine facial lines (Figure 1), hinting at a potential anti-ageing effect on skin. Building on this clinical observation, we employed a D-galactose–induced senescence model in human dermal fibroblasts and found that TXA markedly attenuated cellular senescence and ameliorated senescence-associated phenotypes and molecular markers (Figure 2), thereby confirming a direct anti-ageing action at the cellular level. Taken together, the clinical wrinkle data and the *in vitro* findings suggest that TXA delays senescence by protecting human dermal fibroblasts from oxidative stress, at least in part through G protein–coupled receptor 30 (GPR30)–dependent modulation of the MAPK pathway (Figure 8).

Oxidative stress is a key driver of organismal ageing and many age-related diseases, and the senescence of skin cells is likewise closely linked to the persistent accumulation of reactive oxygen species (ROS) [7–9]. In the present study, under D-galactose–induced oxidative stress, TXA substantially improved the redox status of fibroblasts and reduced oxidative damage (Figure 3), indicating that relief of oxidative stress is a major component of its anti-ageing activity. Sustained oxidative stress not only directly injures DNA, mitochondria and structural proteins, but also activates signalling cascades such as NF-κB and MAPK, thereby initiating and amplifying the senescence-associated secretory phenotype (SASP). Senescent cells then overproduce pro-inflammatory cytokines, chemokines, growth factors and matrix-degrading enzymes, which aggravate cellular senescence and drive chronic tissue inflammation, ultimately establishing a vicious cycle of ‘oxidative stress–SASP–inflammation’ [26–29]. In our experiments, TXA reduced SASP factor expression in senescent fibroblasts, attenuating their amplifying effects on local inflammation and matrix degradation and helping to normalize the chronically inflamed microenvironment characteristic of aged skin (Figure 3). Human dermal fibroblasts are key effector cells in skin ageing and central regulators of the cutaneous inflammatory milieu [2,3]. By secreting pro- and anti-inflammatory mediators, expressing multiple inflammatory receptors and integrating downstream signalling pathways, they participate in the initiation, amplification and resolution of inflammatory responses and play a critical role in the chronic inflammation associated with skin ageing [3,7,10,26]. Their regulation of local inflammatory factors not only shapes their own senescent state and collagen metabolism but also indirectly influences cutaneous immune responses, melanogenesis and dermal matrix homeostasis [2,30]. From this perspective, our data suggest that TXA can mitigate inflammation-associated senescence of fibroblasts by modulating oxidative stress–related pathways.

Previous animal studies have shown that TXA can attenuate age-related changes and prolong lifespan in mice, at least partly by reducing ROS and inflammatory mediators [13]. In physiological models of skin ageing and dryness-induced wrinkling, the improvement in skin parameters with TXA was more pronounced in female mice, and this sex-related difference was associated with elevated plasma 17β-estradiol levels after TXA treatment, implying the involvement of estrogen-related mechanisms [14,31]. Although the mechanism by which TXA promotes estradiol secretion has not been fully clarified, signalling mediated by estrogen receptors is likely to contribute. Our molecular docking results indicate that TXA has a relatively high binding affinity for GPR30, and subsequent cellular experiments confirmed that TXA regulates GPR30 expression, improves oxidative stress status in fibroblasts and thereby exerts anti-ageing effects (Figure 4). Homology modelling and docking studies have suggested that the ligand-binding pocket of GPR30 is predominantly hydrophobic [16,24]. Consistent with this, our docking model indicates that the cyclohexane ring of TXA (its hydrophobic core) can be embedded in this hydrophobic pocket, whereas the side-chain carboxyl (–COOH) and aminomethyl (–CH_2_NH_2_) groups may interact with surrounding amino acid residues. We speculate that under D-galactose–induced oxidative stress, excessive ROS may attack key amino acid residues of GPR30—for example, oxidizing cysteine thiol (–SH) groups to disulfide bonds or converting tyrosine to quinone-like structures—thereby destabilizing receptor conformation, impairing function and accelerating degradation. By contrast, binding of TXA to GPR30 in this context may stabilize the receptor and reduce its degradation. This hypothesis remains to be tested in dedicated structural and biochemical studies.

The mitogen-activated protein kinase (MAPK) cascade is a central signalling hub linking oxidative stress to inflammatory responses and cellular senescence, and represents an important pathway through which ROS have been shown to drive skin ageing [7,10,32]. Previous work has suggested that GPR30 can regulate MAPK activity in various cell types, for example *via* transactivation of the epidermal growth factor receptor (EGFR) [16,18,21,33]. In our system, D-galactose markedly enhanced MAPK activation, whereas TXA pretreatment suppressed this stress-related hyperactivation. When a GPR30 antagonist was applied or GPR30 was knocked down, the inhibitory effect of TXA on MAPK signalling was clearly blunted, accompanied by a rebound in oxidative stress and re-elevation of senescence markers (Figures 5 and 6). Contrary to the conventional view that GPR30 primarily promotes MAPK activation, we observed that, under oxidative stress conditions, TXA modulated GPR30 in a manner associated with reduced MAPK activity and delayed senescence, suggesting that TXA may act as a biased regulator of the GPR30–MAPK axis. Chemically, TXA does not contain classical free radical–scavenging moieties, and its carboxyl and aminomethyl groups are not efficient ROS scavengers, which is consistent with the fact that TXA has not traditionally been regarded as an antioxidant [34,35]. It is therefore reasonable to infer that TXA does not mainly function as a direct chemical scavenger of ROS, but rather as a biased modulator of GPR30, preferentially enhancing antioxidant or negative-feedback pathways downstream of this receptor and ultimately suppressing ROS-driven MAPK activation and cellular senescence. This interpretation is in line with reports that GPR30 can mediate activation of antioxidant pathways such as Nrf2 or AMPK–Nrf2 and thereby alleviate oxidative stress and inflammation in other tissues [16–19,36,37], as well as studies showing that other agents (including genistein, ligustilide and puerarin) protect tissues from injury and photoageing by regulating GPR30/MAPK or related pathways [32,38–40].

This study has several limitations. Although our data show that TXA modulates the expression of several matrix metalloproteinases, suggesting a possible impact on dermal ECM remodelling, we did not directly measure collagen or elastin content in human skin. Future studies that quantify ECM components *in vivo* will be required to determine whether TXA functionally preserves or restores dermal matrix structure. Studies in naturally aged mice have reported that TXA increases dermal collagen content and improves dermal architecture [14,16,31], findings that are broadly consistent with the MMP downregulation and amelioration of senescence phenotypes observed here. Even so, our results demonstrate that TXA alleviates oxidative stress–induced senescent damage in human dermal fibroblasts by regulating GPR30-related signalling, in parallel with measurable improvements in early clinical features of facial ageing. Taken together, these data provide new mechanistic support for the established dermatologic uses of TXA and suggest that TXA may serve as a candidate anti-ageing agent that complements existing topical or systemic strategies for the prevention and treatment of skin ageing.

## Conclusion

In conclusion, topical 3% tranexamic acid was associated with improvement in early periorbital wrinkles in women with facial melasma, and tranexamic acid protected human dermal fibroblasts from D-galactose-induced senescence *in vitro*. These findings suggest that TXA may represent a promising candidate for skin-ageing intervention, at least partly through GPR30-dependent modulation of oxidative stress, SASP-related gene expression, and MAPK signaling.

## Supplementary Material

Supplementary table S1 and S2.docx

## Data Availability

The data that support the findings of this study are available from the corresponding author uponreasonable request.

## References

[1] López-Otín C, Blasco MA, Partridge L, et al. Hallmarks of aging: an expanding universe. Cell. 2023;186(2):243–278. doi:10.1016/j.cell.2022.11.001. 36599349

[2] Solé-Boldo L, Raddatz G, Schütz S, et al. Single-cell transcriptomes of the human skin reveal age-related loss of fibroblast priming. Commun Biol. 2020;3(1):188. doi:10.1038/s42003-020-0922-4. 32327715 PMC7181753

[3] Wlaschek M, Maity P, Makrantonaki E, et al. Connective tissue and fibroblast senescence in skin aging. J Invest Dermatol. 2021;141(4S):985–992. doi:10.1016/j.jid.2020.11.010. 33563466

[4] Franco AC, Aveleira C, Cavadas C. Skin senescence: mechanisms and impact on whole-body aging. Trends Mol Med. 2022;28(2):97–109. doi:10.1016/j.molmed.2021.12.003. 35012887

[5] Azman KF, Zakaria R. D-galactose-induced accelerated aging model: an overview. Biogerontology. 2019;20(6):763–782. doi:10.1007/s10522-019-09837-y. 31538262

[6] Umbayev B, Askarova S, Almabayeva A, et al. Galactose-induced skin aging: the role of oxidative stress. Oxid Med Cell Longev. 2020;2020:7145656–7145615. doi:10.1155/2020/7145656. 32655772 PMC7317321

[7] Papaccio F, D′Arino A, Caputo S, et al. Focus on the contribution of oxidative stress in skin aging. Antioxidants. 2022;11(6):1121. doi:10.3390/antiox11061121. 35740018 PMC9220264

[8] Krutmann J, Schikowski T, Morita A, et al. Environmentally-induced (extrinsic) skin aging: exposomal factors and underlying mechanisms. J Invest Dermatol. 2021;141(4S):1096–1103. doi:10.1016/j.jid.2020.12.011. 33541724

[9] Varesi A, Chirumbolo S, Campagnoli LIM, et al. The role of antioxidants in the interplay between oxidative stress and senescence. Antioxidants. 2022;11(7):1224. doi:10.3390/antiox11071224. 35883714 PMC9311946

[10] Liu H-M, Cheng M-Y, Xun M-H, et al. Possible mechanisms of oxidative stress-induced skin cellular senescence, inflammation, and cancer and the therapeutic potential of plant polyphenols. Int J Mol Sci. 2023;24(4):3755. doi:10.3390/ijms24043755. 36835162 PMC9962998

[11] Forbat E, Al-Niaimi F, Ali FR. The emerging importance of tranexamic acid in dermatology. Clin Exp Dermatol. 2020;45(4):445–449. doi:10.1111/ced.14115. 31663643

[12] Kim KM, Lim HW. The uses of tranexamic acid in dermatology: a review. Int J Dermatol. 2023;62(5):589–598. doi:10.1111/ijd.16160. 35323992

[13] Hiramoto K, Yamate Y, Sugiyama D, et al. Effect of tranexamic acid in improving the lifespan of naturally aging mice. Inflammopharmacology. 2019;27(6):1319–1323. doi:10.1007/s10787-019-00616-2. 31236768

[14] Hiramoto K, Sugiyama D, Iizuka Y, et al. Sex differences regarding the amelioration of wrinkles due to skin dryness by the administration of tranexamic acid. Biomed Pharmacother. 2016;83:283–289. doi:10.1016/j.biopha.2016.06.043. 27393926

[15] Hiramoto K, Yamate Y, Matsuda K, et al. Tranexamic Acid Improves Memory and Learning Abilities in Aging Mice. J Exp Pharmacol. 2020;12:653–663. doi:10.2147/JEP.S284532. 33376415 PMC7755347

[16] Prossnitz ER, Barton M. G protein-coupled oestrogen receptor GPER in health and disease: an update. Nat Rev Endocrinol. 2023;19(7):407–424. doi:10.1038/s41574-023-00822-7. 37193881 PMC10187525

[17] Yasuda H, Sonoda A, Yamamoto M, et al. 17-β-estradiol enhances neutrophil extracellular trap formation by interaction with estrogen membrane receptor. Arch Biochem Biophys. 2019;663:64–70. doi:10.1016/j.abb.2018.12.028. 30590021

[18] Sharma G, Hu C, Staquicini DI, et al. Preclinical efficacy of the GPER-selective agonist G-1 in mouse models of obesity and diabetes, Sci. Sci Transl Med. 2020;12(528):eaau5956. doi:10.1126/scitranslmed.aau5956. 31996464 PMC7083206

[19] Harding AT, Goff MA, Froggatt HM, et al. GPER1 is required to protect fetal health from maternal inflammation. Science. 2021;371(6526):271–276. doi:10.1126/science.aba9001. 33446553 PMC8060949

[20] Meyer MR, Rosemann T, Barton M, et al. GPER mediates functional endothelial aging in renal arteries. Pharmacology. 2017;100(3-4):188–193. doi:10.1159/000478732. 28704834 PMC5558788

[21] Peng J, Zuo Y, Huang L, et al. Activation of GPR30 with G1 attenuates neuronal apoptosis via src/EGFR/stat3 signaling pathway after subarachnoid hemorrhage in male rats. Exp Neurol. 2019;320:113008. doi:10.1016/j.expneurol.2019.113008. 31295444

[22] Lin X, Li L, Wu S, et al. Activation of GPR30 promotes osteogenic differentiation of MC3T3-E1 cells: an implication in osteoporosis. IUBMB Life. 2019;71(11):1751–1759. doi:10.1002/iub.2118. 31298483

[23] Castleman MJ, Pokhrel S, Triplett KD, et al. Innate sex bias of staphylococcus aureus skin infection is driven by α-hemolysin. J Immunol. 2018;200(2):657–668. doi:10.4049/jimmunol.1700810. 29222165 PMC5760295

[24] Arterburn JB, Prossnitz ER. G protein-coupled estrogen receptor GPER: molecular pharmacology and therapeutic applications. Annu Rev Pharmacol Toxicol. 2023;63(1):295–320. doi:10.1146/annurev-pharmtox-031122-121944. 36662583 PMC10153636

[25] Zhang Y, Li L, Xu Y, et al. Protective mechanism of GPR30 agonist G1 against ultraviolet B-induced injury in epidermal stem cells. Artif Cells Nanomed Biotechnol. 2019;47(1):4165–4171. doi:10.1080/21691401.2019.1687497. 31713438

[26] Robbins PD, Jurk D, Khosla S, et al. Senolytic drugs: reducing senescent cell viability to extend health span. Annu Rev Pharmacol Toxicol. 2021;61(1):779–803. doi:10.1146/annurev-pharmtox-050120-105018. 32997601 PMC7790861

[27] Domaszewska-Szostek A, Puzianowska-Kuźnicka M, Kuryłowicz A. Flavonoids in skin senescence prevention and treatment. Int J Mol Sci. 2021;22(13):6814. doi:10.3390/ijms22136814. 34201952 PMC8267725

[28] Wang B, Han J, Elisseeff JH, Demaria M. The senescence‑associated secretory phenotype and its physiological and pathological implications. Nat Rev Mol Cell Biol. 2024;25:958–978. 10.1038/s41580-024-00727-x 38654098

[29] Sun J, Guo Y, Fan Y, et al. Decreased expression of IDH1 by chronic unpredictable stress suppressed proliferation and accelerated senescence of granulosa cells through ROS activated MAPK signaling pathways, Free. Free Radic Biol Med. 2021;169:122–136. doi:10.1016/j.freeradbiomed.2021.04.016. 33865962

[30] Chen M, Yang L, Zhou P, et al. Single-cell transcriptomics reveals aberrant skin-resident cell populations and identifies fibroblast signature as a key determinant in rosacea. Nat Commun. 2024;15(1):8737. doi:10.1038/s41467-024-52946-7. 39384741 PMC11464544

[31] Hiramoto K, Yamate Y, Sugiyama D, et al. Ameliorative effect of tranexamic acid on physiological skin aging and its sex difference in mice. Arch Dermatol Res. 2019;311(7):545–553. doi:10.1007/s00403-019-01938-5. 31147768

[32] Kim M, Kim J, Jeong GJ, et al. Particulate matter induces pro-inflammatory cytokines via phosphorylation of p38 MAPK possibly leading to dermal inflammaging. Exp Dermatol. 2019;28(7):809–815. doi:10.1111/exd.13943. 31001893

[33] Prossnitz ER, Arterburn JB, Smith HO, et al. Estrogen signaling through the transmembrane G protein-coupled receptor GPR30. Annu Rev Physiol. 2008;70(1):165–190. doi:10.1146/annurev.physiol.70.113006.100518. 18271749

[34] Goobie SM. Tranexamic acid: still far to go. Br J Anaesth. 2017;118(3):293–295. doi:10.1093/bja/aew470. 28203768

[35] Barrett CD, Kong YW, Yaffe MB. Influence of Tranexamic Acid on inflammatory Signaling in Trauma. Semin Thromb Hemost. 2020;46(2):183–188. doi:10.1055/s-0040-1702169. 32160643

[36] Yin J, Zhang Y, Liu X, et al. Gut microbiota-derived indole derivatives alleviate neurodegeneration in aging through activating GPR30/AMPK/SIRT1 pathway. Mol Nutr Food Res. 2023;67(9):e2200739. doi:10.1002/mnfr.202200739. 36823436

[37] Yao Y, Wang H, Yang Y, et al. Dehydroepiandrosterone protects against oleic acid-triggered mitochondrial dysfunction to relieve oxidative stress and inflammation via activation of the AMPK-nrf2 axis by targeting GPR30 in hepatocytes. Mol Immunol. 2023;155:110–123. doi:10.1016/j.molimm.2023.01.008. 36773597

[38] Yang F, Lin ZW, Huang TY, et al. Ligustilide, a major bioactive component of Angelica sinensis, promotes bone formation via the GPR30/EGFR pathway. Sci Rep. 2019;9(1):6991. doi:10.1038/s41598-019-43518-7. 31061445 PMC6502875

[39] Wang J-Y, Xie X-Y, Deng Y, et al. Licorice zinc suppresses melanogenesis via inhibiting the activation of P38MAPK and JNK signaling pathway in C57BL/6J mice skin. Acta Cir Bras. 2022;37(10):e371002. doi:10.1590/acb371002. 36542040 PMC9762428

[40] Mo Q, Li S, You S, et al. Puerarin reduces oxidative damage and photoaging caused by UVA radiation in human fibroblasts by regulating nrf2 and MAPK signaling pathways. Nutrients. 2022;14(22):4724. doi:10.3390/nu14224724. 36432411 PMC9694396

