# Supplementary material for: Tranexamic acid protects human dermal fibroblasts from D-galactose-induced senescence via the GPR30/MAPK pathway
Source: Ann Med. 2026 Apr 30;58(1):2663263. doi: 10.1080/07853890.2026.2663263 (PMC13134749; doi:10.1080/07853890.2026.2663263)
Supplement: Supplementary table S1 and S2.docx [file IANN_A_2663263_SM6240.docx]

**Table S1. Antibodies used for Western blotting in this study**

| **Target protein** | **Antibody name 2** | **Manufacturer** | **Catalogue No.** | **Host / type** | **Application** | **Working dilution**  **(WB)** |
| --- | --- | --- | --- | --- | --- | --- |
| p21 | p21 Waf1/Cip1 (12D1) Rabbit mAb | Cell Signaling Technology | #2947 | Rabbit monoclonal | WB | 1:1000 |
| p16 | p16INK4a Antibody | Cell Signaling Technology | #4824 | Rabbit polyclonal | WB | 1:1000 |
| pRb | Phospho-Rb (Ser807/811) (D20B12) XP Rabbit mAb | Cell Signaling Technology | #8516 | Rabbit monoclonal | WB | 1:1000 |
| Rb | Rb (4H1) Mouse mAb | Cell Signaling Technology | #9309 | Mouse monoclonal | WB | 1:2000 |
| Lamin B1 | Lamin B1 (D4Q4Z) Rabbit mAb | Cell Signaling Technology | #12586 | Rabbit monoclonal | WB | 1:1000 |
| Phospho-JNK (p-JNK) | Phospho-SAPK/JNK (Thr183/Tyr185) Antibody | Cell Signaling Technology | #9251 | Rabbit polyclonal | WB | 1:1000 |
| JNK | SAPK/JNK Antibody | Cell Signaling Technology | #9252 | Rabbit polyclonal | WB | 1:1000 |
| Phospho-ERK (p-ERK) | Phospho-p44/42 MAPK (Erk1/2) (Thr202/Tyr204) Antibody | Cell Signaling Technology | #9101 | Rabbit polyclonal | WB | 1:1000 |
| ERK | p44/42 MAPK (Erk1/2) Antibody | Cell Signaling Technology | #9102 | Rabbit polyclonal | WB | 1:1000 |
| Phospho-p38 (p-p38) | Phospho-p38 MAPK (Thr180/Tyr182) Antibody | Cell Signaling Technology | #9211 | Rabbit polyclonal | WB | 1:1000 |
| p38 | p38 MAPK Antibody | Cell Signaling Technology | #9212 | Rabbit polyclonal | WB | 1:1000 |
| GPR30 | Anti-G-protein coupled receptor 30 (GPR30/GPER1) antibody | Abcam | ab39742 | Rabbit polyclonal | WB | 1:1000 |

**Table S2**. qPCR Primers

| Gene name | Sequence 5′-3′ (Forward) | Sequence 5′-3′ (Reverse) |
| --- | --- | --- |
| GPR30 | CACCAGCAGTACGTGATCGG | CATCTTCTCGCGGAAGCTGAT |
| IL-6 | CCTGAACCTTCCAAAGATGGC | TTCACCAGGCAAGTCTCCTCA |
| IL-8 | TTCTGCAGCTCTGTGTGAAG | CCAGTTTTCCTTGGGGTCCA |
| MMP1 | AAAATTACACGCCAGATTTGCC | GGTGTGACATTACTCCAGAGTTG |
| MMP3 | CTGGACTCCGACACTCTGGA | CAGGAAAGGTTCTGAAGTGACC |
| GAPDH | GGAGCGAGATCCCTCCAAAAT | GGCTGTTGTCATACTTCTCATGG |
